# Updates on the Management of Ampullary Neoplastic Lesions

**DOI:** 10.3390/diagnostics13193138

**Published:** 2023-10-06

**Authors:** Roberta Maselli, Roberto de Sire, Alessandro Fugazza, Marco Spadaccini, Matteo Colombo, Antonio Capogreco, Torsten Beyna, Alessandro Repici

**Affiliations:** 1 Gastroenterology, Endoscopy Unit, IRCCS Humanitas Research Hospital, 20089 Rozzano, Italy; roberta.maselli@hunimed.eu (R.M.); alessandro.fugazza@humanitas.it (A.F.); marco.spadaccini@humanitas.it (M.S.); matteo.colombo@humanitas.it (M.C.); antonio.capogreco@humanitas.it (A.C.); alessandro.repici@hunimed.eu (A.R.); 2 Department of Biomedical Sciences, Humanitas University, 20072 Pieve Emanuele, Italy; 3 Gastroenterology Unit, Department of Clinical Medicine and Surgery, University Federico II of Naples, 80126 Naples, Italy; 4 Department of General Internal Medicine and Gastroenterology, Evangelisches Krankenhaus, 40217 Düsseldorf, Germany; torsten.beyna@evk-duesseldorf.de

**Keywords:** ampullary neoplastic lesion, ampullary tumor, endoscopic papillectomy, ampullectomy, ERCP

## Abstract

Ampullary neoplastic lesions (ANLs) represent a rare cancer, accounting for about 0.6–0.8% of all gastrointestinal malignancies, and about 6–17% of periampullary tumors. They can be sporadic or occur in the setting of a hereditary predisposition syndrome, mainly familial adenomatous polyposis (FAP). Usually, noninvasive ANLs are asymptomatic and detected accidentally during esophagogastroduodenoscopy (EGD). When symptomatic, ANLs can manifest differently with jaundice, pain, pancreatitis, cholangitis, and melaena. Endoscopy with a side-viewing duodenoscopy, endoscopic ultrasound (EUS), and magnetic resonance cholangiopancreatography (MRCP) play a crucial role in the ANL evaluation, providing an accurate assessment of the size, location, and characteristics of the lesions, including the staging of the depth of tumor invasion into the surrounding tissues and the involvement of local lymph nodes. Endoscopic papillectomy (EP) has been recognized as an effective treatment for ANLs in selected patients, providing an alternative to traditional surgical methods. Originally, EP was recommended for benign lesions and patients unfit for surgery. However, advancements in endoscopic techniques have broadened its indications to comprise early ampullary carcinoma, giant laterally spreading lesions, and ANLs with intraductal extension. In this paper, we review the existing evidence on endoscopic diagnosis and treatment of ampullary neoplastic lesions.

## 1. Introduction

Ampullary neoplastic lesions (ANLs) represent a rare cancer, accounting for about 0.6–0.8% of all gastrointestinal malignancies, and about 6–17% of periampullary tumors (arising from the ampulla of Vater, the distal common bile duct, the second portion of the duodenum, and the head of the pancreas) [1,2,3].

Although the overall incidence of ANLs in western countries is less than 1 case per 100,000 per year according to data from international registries, their incidence has risen during the last decades, probably due to the growing use of esophagogastroduodenoscopy (EGD) for diagnosis of other upper-GI disorders and for the screening of high-risk patients with familial adenomatous polyposis (FAP) [4,5].

ANLs are often sporadic and arise from intestinal-type mucosa, involving the major papilla, and following an adenoma-to-carcinoma sequence with a potential transformation into adenocarcinoma [6]. However, ANLs can originate from different tissues, expressing in a different way compared to the better-known intestinal type of lesions, including the pancreaticobiliary type (from pancreatic duct-type ampullary mucosa), the mixed type (glandular and squamous cell tissue), the mucinous type (colloid), the signet-ring cell carcinomas, the neuroendocrine type, and the undifferentiated type [7]. Historically, intestinal-type ANLs have been associated with a more benign clinical course compared to pancreaticobiliary-type malignancies [8,9]; nevertheless, recent studies have not confirmed this suggestion, finding no prognostic differences between the two groups [10,11].

Hereditary ANLs appear in younger age groups than sporadic ones; among hereditary syndromes that increase ANL predisposition, including the neurofibromatosis type I and the Muir–Torre syndrome, the familial adenomatous polyposis (FAP) is associated with a 120-fold increased relative risk to have an ampullary tumor compared to the general population [12].

## 2. Diagnosis

### 2.1. Clinical Assessment

Usually, noninvasive ANLs are asymptomatic and detected accidentally during EGD performed for another indication. When symptomatic, ANLs can manifest differently with jaundice, pain, diarrhea, pancreatitis, cholangitis, steatorrhea, and melaena, making the differential diagnosis with choledocholithiasis, extrahepatic cholangiocarcinoma, and pancreatic adenocarcinoma [13,14,15]. 

### 2.2. Endoscopy

**Figure 1:**
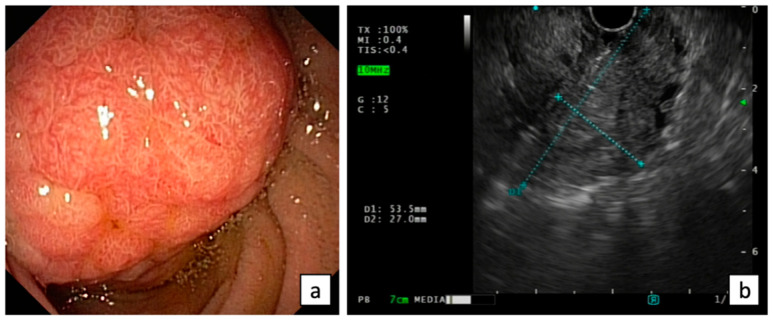
Endoscopic (**a**) and EUS (**b**) evaluation of ANLs.

Endoscopic evaluation of ANLs may be challenging using a forward-viewing endoscope. The European Society of Gastrointestinal Endoscopy (ESGE) considers the visualization of the papilla as a research priority for a complete high-quality endoscopy [16]. Thus, cap-assisted upper endoscopy should be indicated when the major papilla is not seen by a gastroscope, but a side-viewing duodenoscopy is recommended for the optimal visualization of the papilla and the assessment of the feasibility of endoscopic resection in presence of ANLs [17] (Figure 1).

Currently, an endoscopic classification of ANLs is still lacking. They can present as isolated lesions of the papilla, have an extra-papillary component (7–44%), and in some cases an intra-ductal component [18,19,20,21,22,23]. Benign small ANLs can be indistinguishable from normal papilla, while ANLs with laterally spreading growth could have an extra-papillary component showing similar characteristics to non-ampullary duodenal adenomas [22]. Benign features include regularity of surface and margins, soft consistency, and mobility, while superficial erosions, ulcers, friability, hard consistency, firmness, and spontaneous bleeding are usually associated with malignancy [23,24]. Furthermore, large lesions (>20 mm) have been associated with an increased risk of deep invasion at histology and local recurrence after resection in several studies [25,26,27].

### 2.3. Chromoendoscopy

Chromoendoscopy is a valuable tool in the diagnosis ANL, enhancing the visualization of mucosal details and facilitating the differentiation between benign and neoplastic lesions. By evaluating the microsurface and microvessel patterns of lesions, chromoendoscopy can provide insights into the histology and grade of dysplasia, guiding endoscopic diagnosis and management. Indeed, irregular villous arrangement and abnormal microvasculature are evaluated with virtual chromoendoscopy, such as narrow band imaging (NBI), and it diagnoses adenocarcinoma with sensitivity, specificity, positive predictive value, negative predictive value, and accuracy of 69%, 100%, 100%, 85%, and 89%, respectively [28]. Furthermore, dye-based chromoendoscopy with indigo carmine appeared particularly useful in delineating the margins of the lesion prior to endoscopic papillectomy, ensuring complete resection, and reducing the risk of local recurrence [29].

### 2.4. Histology

Endoscopic biopsies and hematoxylin and eosin-stained histopathology are mandatory for the diagnosis of ANLs, although their diagnostic accuracy has been reported to range widely from 38% to 85% [30,31,32,33,34,35]. The histological underestimation rate can reach up to 30% [36,37]. Prospectively evaluated, the overall rate of diagnostic overestimation, which can result in potentially inadequate and risky treatment, stands at 15%, with a specific rate of 21% for initial low-grade dysplasia diagnosis [18]. Furthermore, in two extensive retrospective series, post-papillectomy histological analysis revealed that normal intestinal mucosa or inflammation alone were found in 8% and 13.8%, respectively [35,38].

ESGE recommends obtaining histological confirmation through repeat endoscopic biopsies before initiating any treatment in the presence of low-grade dysplasia [19]. Conversely, if the presence of adenoma has not been established, ESGE does not recommend diagnostic or therapeutic papillectomy [19]. 

Several reports have suggested that in cases of a bulging papilla without features of abnormality, endoscopic biopsies should be performed subsequent to an endoscopic sphincterotomy. Nevertheless, conflicting findings have been documented, revealing low sensitivities ranging from 21% to 37% due to the potential occurrence of cytoarchitectural atypia resulting from post-sphincterotomy changes [39,40]. In this context, performing additional samplings, preferably at least 10 days after sphincterotomy, can prove beneficial in order to prevent initial false-negative results [41].

### 2.5. Immunohistochemistry, Polymerase Chain Reaction, and Flow Cytometry

Presently, it is not recommended to routinely employ immunohistochemical staining for the p53 tumor suppressor gene, polymerase chain reaction analysis of tumor DNA for K-ras gene mutations, and the addition of flow cytometry to assess aneuploidy for prognosis determination and/or prediction of treatment response. However, the subclassification of ANLs into intestinal or pancreaticobiliary phenotypes holds considerable prognostic significance. In resected specimens, immunohistochemistry (IHC) panels comprising MUC1, MUC2, CDX2, CK20, and MUC5AC can assist in subtyping into intestinal or pancreaticobiliary phenotypes [42,43]. However, for endoscopic biopsies, the morphological and IHC classifications into intestinal or pancreaticobiliary phenotypes lack consistency due to factors such as tissue heterogeneity, antigenicity, interpretation of staining patterns, and inter/intraobserver variability. The presence of K-ras and p53 mutations has been identified in various histological subtypes of ANLs. However, these mutations do not provide a definitive histological subtyping of intestinal and pancreaticobiliary phenotypes, highlighting the frequent occurrence of hybrid phenotypes [44].

### 2.6. EUS, MRCP, and IDUS

Endoscopic ultrasound (EUS) plays a crucial role in the ANL evaluation, providing an accurate assessment of the size, location, and characteristics of the lesions, including the staging of the depth of tumor invasion into the surrounding tissues such as duodenal wall, biliary duct, pancreatic duct, and pancreatic parenchyma, and the involvement of local lymph nodes, according to the latest TNM classification (Figure 1).

The diagnostic accuracy of endoscopic ultrasound EUS for detecting tumor depth (T-staging) and regional lymph node status (N-staging) was evaluated in a recent meta-analysis that included 21 studies [45]. The pooled sensitivity and specificity of EUS were 0.89 and 0.87 for T1, 0.76 and 0.91 for T2, 0.81 and 0.94 for T3, and 0.72 and 0.98 for T4, respectively. For N-staging, 16 studies using EUS were included with sensitivity and specificity of 0.61 and 0.77, respectively [45]. When compared with magnetic resonance cholangiopancreatography (MRCP), EUS demonstrates comparable or slightly higher accuracy for T staging, while for N staging, MRCP had the best performance [46,47,48,49,50]. 

Among intraductal biliopancreatic imaging techniques, intraductal ultrasonography (IDUS) offers real-time, cross-sectional imaging of the pancreatobiliary ducts and nearby structures while performing ERCP using a high-frequency ultrasound transducer. Consequently, IDUS is highly regarded as a sensitive and valuable tool for evaluating ANLs. According to Ye et al. The pooled sensitivity and specificity of IDUS were 0.90 and 0.88 for T1, 0.73 and 0.91 for T2, and 0.79 and 0.97 for T3, respectively. Considering N-staging, the pooled sensitivity and specificity of IDUS were 0.61 and 0.92, respectively [45]. However, IDUS is now regarded as an outdated technique that is no longer used mainly due to its high costs, the fragility of the devices, and the complexity of the training required to learn it. 

## 3. Treatment

### 3.1. Endoscopic Treatment

**Figure 2:**
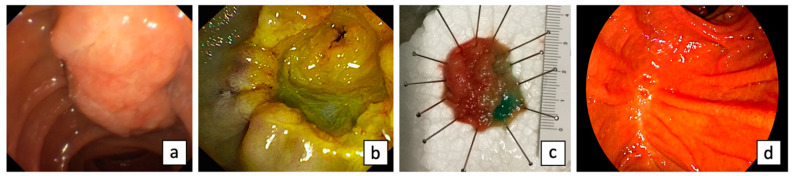
Endoscopic papillectomy: ANL evaluated by side-viewing duodenoscopy (**a**); post-EP inspection (**b**); specimen (**c**); and scar at 1-year follow-up (**d**).

Endoscopic papillectomy (EP) refers to the endoscopic resection of the mucosa and submucosa of the duodenal wall, including the region where the ampulla of Vater is anatomically attached, including the excision of the surrounding tissue around the orifices of the bile duct and pancreatic duct [51] (Figure 2). First described in 1983 by Suzuki et al. [52], endoscopic papillectomy (EP) has been recognized as an effective treatment for ANLs in selected patients, providing an alternative to traditional surgical methods [53]. Originally, EP was recommended for benign lesions and patients unfit for surgery [53]. However, advancements in endoscopic techniques have broadened its indications to comprise early ampullary carcinoma, giant laterally spreading lesions, and ANLs with intraductal extension [53].

The primary purpose of submucosal injection for ANLs before EP is to assist in diagnosing the lateral extent of the lesion. Additionally, a no lifting sign may indicate the presence of deep submucosal invasion that cannot be effectively treated through a conventional endoscopic resection. Submucosal injection is also advocated to prevent bleeding and reduce the risk of causing deep thermal damage to the ducts and muscularis propria during the procedure. Nevertheless, according to a survey conducted with 46 expert biliary endoscopists in the USA and Canada, the use of submucosal injection in combination with EP does not appear to offer any significant advantage [54]. Currently, there is only one randomized controlled trial that has compared EP with or without submucosal injection [55]. The complete resection rate was significantly higher in the no-injection group compared to the injection group (80.8% vs. 50.0%, respectively; *p* = 0.02). However, there were no notable differences in terms of AEs, residual tumor at 1 month, and local recurrence at 12 months [55]. 

Although there is a lack of clear evidence from comparative trials, many authors recommend performing a cholangiogram and pancreatogram prior to EP to assess for deep intraductal extension exceeding 10 mm. The routine use of bi-ductal sphincterotomy before resection granted comparable technical and clinical success to the standard technique with a low adverse event (AE) rate of 8% [56]. However, it is worth noting that the number of en bloc and single-session resections appears to be lower, especially when sphincterotomy is combined with pancreatic stent placement before resection. Furthermore, it has been shown that altered papilla morphology is associated with a higher risk of biliary cannulation failure and AEs [57]. Additionally, some authors have reported challenges in obtaining a complete histopathological evaluation of the resected specimen due to thermal injury following sphincterotomy [58,59].

There is currently no consensus regarding the optimal current and power output for performing endoscopic papillectomy. In an RCT conducted by Iwasaki et al. [60], it was demonstrated that both autocut and endocut mode exhibit similar efficacy and safety for EP. However, the endocut mode may offer an advantage by potentially preventing immediate bleeding in cases involving large tumor sizes (88% vs. 46%, *p* = 0.04), although the rate of crush artifacts was higher in the endocut group compared to the autocut group (27% vs. 3.3%, *p* = 0.03). 

According to a recent systematic review with pooled analysis, that included 29 studies reporting the results of EP in a total of 1751 patients with ANLs, complete endoscopic resection was achieved in 94.2% patients (95 %CI 90.5–96.5; I2 = 73%), and curative endoscopic resection in 87.1% patients (95 %CI 83.0–90.3; I2 = 70%) [25]. En bloc resection was achieved in 82.4 % (95 %CI 74.7–88.1; I2 = 84%), and this was the only factor affecting curative resection (odds ratio [OR] = 3.55, 95 %CI = 1.11–5.99, *p* = 0.004) [25]. The overall rate of AEs following EP was 24.9% (95% CI = 21.2%–29.0%; I2 = 66%) [25]. The most frequently reported AEs were post-procedural pancreatitis, occurring in 11.9% of cases (95% CI 10.4–13.6; I2 = 41%), followed by bleeding in 10.6% cases (95% CI = 5.2–13.6; I2 = 61%) [25]. Perforations and cholangitis were less common, reported in 3.1% (95% CI = 2.2–4.2; I2 = 17%) and 2.7% (95% CI = 1.9–4.0; I2 = 32%) of cases, respectively [25]. The occurrence of long-term AEs such as papillary stenosis was recorded in 2.4% of cases (95% CI = 1.6–3.4; I2 = 0). The mortality rate associated with the procedure was 0.3% [25]. 

Binda et al. [61] provided data on the effectiveness and safety of EP in a multicenter, retrospective, nationwide study, including a total of 225 ANL patients. En bloc resection was possible in 72.5% of cases, with an overall R0 resection rate of 50.7% [61]. During a mean follow-up period of 23.2 months, recurrences were diagnosed in 17.2% of patients, 61.3% of which were successfully treated with an additional endoscopic treatment with a clinical success achieved in 76.7% of the cases [61]. In multivariate analysis, R1 resection, lesion size, and histological diagnosis were predictors for recurrence. Intra-procedural bleeding occurred during 12.4% of EP [61]. Post-EP AEs occurred in 39.5% of patients, including delayed bleeding (20.9%), pancreatitis (13.3%), and perforation (2.2%) [61].

Among novel resection techniques of ANL, Takahara et al. [62] evaluated a modified EP with hybrid endoscopic submucosal dissection (hybrid ESD-EP), consisting of a (sub)circumferential incision with partial submucosal dissection, and subsequent snare resection, in order to attain a higher en bloc resection rate with curative safe margin compared to the standard technique. En bloc resection was achieved with hybrid ESD-EP in all eight cases (100%), with all lateral margins clear (100%), whereas vertical margin was uncertain in three (38%), resulting in the complete resection rate of 63% [62]. Post-operative bleeding and pancreatitis developed in 13% of cases [62]. After a median follow-up of 9 months, no tumor recurrence was observed even in those cases with uncertain vertical margin [62]. A summary of the more recent results described in the literature is provided in Table 1.

EP has proven to be a valuable treatment option also for FAP-related ANLs. Despite the effectiveness and safety of EP in treating FAP-related ANLs, it is important to note that FAP patients have a lifetime risk of relapse even after achieving complete resection. Consequently, long-term surveillance is necessary to closely monitor these patients. Recently, in a retrospective multicenter study involving 1422 endoscopic papillectomy procedures, a propensity score matching approach was employed [63]. The matching process considered factors such as age, sex, comorbidity, histologic subtype, and size [63]. The purpose was to analyze main outcomes, including complete resection (R0), technical success, complications, and recurrence, within matched cohorts of FAP-related and sporadic ANLs [63]. Among the FAP patients, the majority (79.2%; 95% CI = 71.2–87.3) were asymptomatic, which was significantly higher compared to the sporadic group (46.5%; 95% CI = 36.6–56.4; *p* < 0.001) [63]. The initial rate of complete resection (R0) was significantly lower in FAP patients (63.4%; 95% CI = 53.8–72.9) compared to controls (83.2%; 95% CI = 75.8–90.6; *p* = 0.001) [63]. However, after subsequent interventions (mean of 1.30 interventions per patient), the R0 rates became comparable between the two groups (FAP: 93.1%; 95% CI = 88.0–98.1 vs. sporadic: 97.0%; 95% CI = 93.7–100; *p* = 0.19) [63]. Adverse events were observed in 28.7% of cases, with pancreatitis and bleeding being the most common in both FAP and sporadic groups [63]. Severe AEs were rare, occurring in only 3.5% of cases [63]. Recurrence was observed in 21 FAP patients (20.8%; 95% CI = 12.7–28.8) and 16 sporadic patients (15.8%; 95% CI = 8.6–23.1; *p* = 0.36) [63]. However, recurrences were noted to occur later in FAP patients, with a median time of 25 months (95% CI = 18.3–31.7), compared to 2 months (95% CI = 0.06–3.9) in the control group [63].

Regarding laterally spreading tumors involving the papilla of Vater (LST-p), characterized as an ampullary tumor that extends laterally beyond the ampullary mound by ≥10 mm [22], or has an extra-papillary component on the duodenal wall larger than the size of the ANL [64], in several retrospective cohorts, it was shown that the endoscopic treatment has comparable outcomes in terms of endoscopic curative resection and recurrence rates, when compared to ANLs [19,22,64,65]. However, Klein et al. [22] have reported a higher risk of intraprocedural bleeding (50% vs. 24.7%, *p* = 0.003), as well as delayed bleeding (25% vs. 12.3%, *p* = 0.08) with LST-p. Similar findings were observed by Sahar et al. [64] in terms of delayed bleeding (14% vs. 4%, *p* = 0.02). However, cold snaring can lead to significant safety over hot snare-based techniques for treatment of the duodenal extra-papillary component such as for superficial non-ampullary duodenal epithelial tumors, without an impairment in terms of curability [66]. Indeed, as reported by Repici et al. in a retrospective multicenter study comparing 33 large duodenal adenomas treated with cold-snaring to 101 patients who had hot EMR, no serious AEs occurred in the cold group, while 17 intraprocedural serious AEs (16.8%) and 26 postprocedural serious AEs (25.7%) in the hot EMR group occurred with a local recurrence at first follow-up endoscopy comparable in both groups (cold EMR: 4/33; 12.1% versus hot EMR: 21/101; 20.8%) [67].

In cases of intraductal growth, Bohnacker et al. [68] have reported a lower rate of endoscopic curative resection (46% vs. 83%, *p* < 0.001) and a higher rate of rescue surgery (37% vs. 12%) compared with ANLs without intraductal extension. Nevertheless, ESGE suggests the use, in tertiary centers, of complementary techniques, including thermal ablation with cystotome, or radio-frequency ablation (RFA) with temporary biliary stenting, for ANLs with ≤20 mm intraductal extension [19]. In a retrospective observational study, Pérez-Cuadrado-Robles et al. [23] proposed the use of endoscopic thermal ablation performed with a wire-guided cystotome and soft/forced coagulation, obtaining an intraductal ablation in 100% of cases with a 20-month median follow-up. Furthermore, in a recent randomized controlled trial, involving 20 patients who had undergone endoscopic papillectomy for ampullary adenoma and were found to have histologically proven endobiliary adenoma remnants (with a ductal extent <20 mm), it was found that intraductal RFA achieved a 70% eradication of dysplasia at 12 months following a single session [69]. Endoscopic ID-RFA showed good long-term outcomes in treating residual or relapsed ANLs with intraductal extension, and repeated ID-RFA may be considered as an option for managing recurrence [70].

### 3.2. Management of Adverse Events

According to the literature, EP shows a lower overall adverse events (AEs) rate compared to surgical treatment. However, it is essential to note that even after EP, a significant AEs rate persists. An overall AEs rate of 24.9% (95% CI, 21.2% to 29%) was reported, but many complications are typically mild to moderate and can be managed conservatively [25]. AEs can be classified into two categories: early AEs, including pancreatitis, bleeding, and perforation, and delayed AEs, such as papillary and biliary stenosis or duodenal luminal stenosis. 

Postprocedural pancreatitis, caused by the obstruction of the pancreatic orifice because of the transient edema from the electrocautery, has been reported to be the most common adverse event occurring in 11.9% of cases (95% CI, 10.5% to 13.6%) [25]. Prophylactic pancreatic duct stenting is recommended as an effective technique for prevention of pancreatitis after EP [17]. Indeed, a single RCT revealed a significantly lower incidence of pancreatitis (33%) in the stented group compared to the unstented group (*p* = 0.02) [71], while the only factor leading to acute pancreatitis as an outcome was the placement of a prophylactic pancreatic stent during the same session (OR −1.72, 95% CI −2.95 to −0.50; *p* = 0.006), as described in a systematic review with pooled analysis [25]. 

In this context, biodegradable pancreatic stents offer the promise of eliminating the need for repetitive endoscopies to remove stents, diminishing potential patient risks, and cutting down on healthcare expenses [72]. Furthermore, to prevent complications, it is recommended to administer 100 mg of rectal indomethacin or diclofenac immediately before EP in all patients without any contraindications [17]. The confirmation of a pancreas divisum, documented during the preoperative EUS or MRCP, avoids the need for pancreatic stent placement [17].

Bleeding is the second most frequent complication (10.6%; 95% CI, 5.2% to 13.6%) [25]. Bleeding can be distinguished in intraprocedural or delayed, usually in the first 12 h after resection [73]. In the more recent and largest retrospective studies, bleeding management was performed endoscopically in 69.1% of cases (n = 56/81) and mostly successfully [19,36,38,64,74]. If bleeding occurs, there are several approaches to manage it. One option is to attempt epinephrine injection, but also soft coagulation using the tip of the snare or coagulation forceps; alternatively, the use of APC (Argon Plasma Coagulation) or the application of clips can be considered [73]. In cases of endoscopic failure, an angiographic evaluation and embolization could be considered [19,36,38,65,74].

**Table 1:** Main outcomes of endoscopic papillectomy.

First Author, Year	Subjects, n, Study Design	Outcomes, n, n (%)			
		En Bloc	Clinical Success	Overall AEs	Local Recurrence
Binda, 2023 [61]	225, Retrospective Study	163/225 (72.5%)	173/225 (76.7%)	89/225 (39.5%)	39/225 (17.2%)
Takahara, 2020 [62]	8, Retrospective Study	8/8 (100%)	8/8 (100%)	6/8 (75%)	0/8 (0%)
Sahar, 2020 [64]	161, Retrospective Study	115/161 (72%)	106/128 (83%)	24/161 (14.9%)	12/161 (7%)
Tringali, 2020 [74]	135, Retrospective Study	112/135 (83%)	96/103 (93%)	29/135 (21.5%)	24/103 (23%)
Li, 2019 [36]	110, Retrospective Study	83/110 (75.5%)	86/110 (78.2%)	39/110 (35%)	13/110 (11.8%)
van der Wiel, 2019 [19]	87, Retrospective Study	41/87 (47.1%)	67/87 (77%)	23/87 (26.4%)	10/87 (11.5%)

Perforation related to electrocautery is reported in 3.1% of cases (95% CI, 2.2% to 4.2%) [25]. It is crucial to carefully inspect the defect both endoscopically and fluoroscopically to detect any deep tissue injury. Fortunately, due to its retroperitoneal location, perforation can typically be managed conservatively. In cases where perforation is diagnosed during the procedure, intravenous antibiotics should be administered, and an attempt to close the perforation using endoclips, along with biliary stenting using a fully covered self-expandable metallic stent, is recommended [73]. 

### 3.3. Surgical Treatment

**Figure 3:**
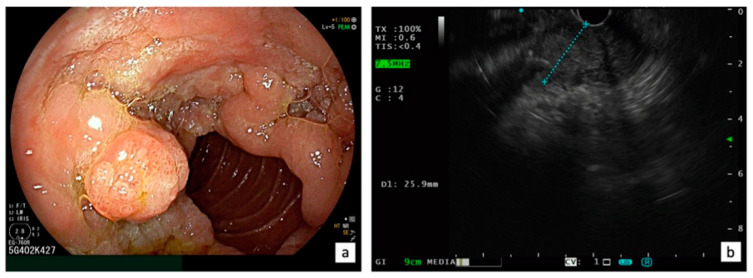
Indication for surgical treatment: LST-p characterized with a circumferential extra-papillary component on the duodenal wall with a tumor size exceeding 4 cm (**a**) and an intraductal involvement >20 mm confirmed using EUS (**b**).

Presently, there is a lack of RCTs that compare EP and surgical treatments, such as transduodenal ampullectomy or pancreaticoduodenectomy, for ANLs. In a recent systematic review and meta-analysis, including a total of 39 studies with 1753 patients (1468 EP and 285 transduodenal ampullectomy), Garg et al. [75] aimed to compare long-term recurrence of benign sporadic ANLs after EP and transduodenal ampullectomy, showing that endoscopic and surgical ampullectomy have similar recurrence rates at 1, 2, 3, and 5 years of follow-up. Thus, when feasible, EP is considered the first choice for ANL. However, there are still situations where surgery remains a viable option for ANL, including the following: (1) intraductal involvement (>20 mm); (2) technical limitations (the presence of a diverticulum or the tumor size exceeding 4 cm); (3) incomplete resection after EP (positive margins); and (4) local recurrence not endoscopically treatable [76] (Figure 3). A systematic review, which included five retrospective cohort studies with 466 patients, revealed that surgical resection (transduodenal ampullectomy or pancreaticoduodenectomy) had better outcomes in achieving complete resection of ANLs compared to EP (risk difference [RD] −0.37, 95% CI −0.50 to −0.24, *p* < 0.001, I 2 = 71%) without any difference in complications [77]. However, when utilizing a fixed effects model, EP exhibited a lower rate of adverse events (RD −0.28, 95% CI −0.39 to −0.18, *p* < 0.001; I 2 = 95%) [77].

The treatment approach for ampullary cancer is pancreaticoduodenectomy, which is characterized by post-operative morbidity ranging from 34% to 59% and a mortality rate of 1% to 2%. After surgery, the 5-year survival rates for ampullary cancer range between 40% and 60% and the main prognostic factors that significantly impact survival are the T and N status of the tumor [26,78,79].

In case of Tis ampullary lesions, transduodenal ampullectomy has shown lower morbidity rates compared to pancreaticoduodenectomy, with no risk of recurrence [80,81,82]. The 5-year survival rate of patients with early ampullary cancer was 77.3% in transduodenal ampullectomy group and 75.9% in pancreaticoduodenectomy group (*p* = 0.927) [82].

Nevertheless, due to a significant percentage of T1 ampullary carcinomas showing lymph node metastasis, with rates ranging from 9% to 45% [26,78,80,83], pancreaticoduodenectomy with lymphadenectomy remains the procedure of choice for T1 adenocarcinoma.

## 4. Conclusions

ANLs represent a rare cancer that can be sporadic or occur in the setting of a hereditary predisposition syndrome. The management of ANLs is hampered by the paucity of data available in the literature, mainly represented by retrospective studies. Endoscopy, EUS, and MRCP play a crucial role in the evaluation and the staging of ANLs, providing an accurate assessment of the size, location, and characteristics of the lesions, and the depth of tumor invasion into the surrounding tissues and the involvement of local lymph nodes. EP has been recognized as an effective treatment for ANLs in selected patients, providing an alternative to traditional surgical methods. Advancements in endoscopic techniques have broadened its indications to comprise early ampullary carcinoma, giant laterally spreading lesions, and ANLs with intraductal extension. Nevertheless, EP remains a complex procedure with an increased risk of AEs. Thus, it is strongly recommended that this procedure be exclusively carried out within proficient medical centers, led by operators skilled in endoscopic resection methods able to effectively manage any associated AEs, ensuring good outcomes and low morbidity rates.
